# Arterial stiffness and its association with clustering of metabolic syndrome risk factors

**DOI:** 10.1186/s13098-017-0286-1

**Published:** 2017-10-25

**Authors:** Wanda R. P. Lopes-Vicente, Sara Rodrigues, Felipe X. Cepeda, Camila Paixão Jordão, Valéria Costa-Hong, Akothirene C. B. Dutra-Marques, Jefferson C. Carvalho, Maria Janieire N. N. Alves, Luiz A. Bortolotto, Ivani C. Trombetta

**Affiliations:** 1 Universidade Nove de Julho (UNINOVE), Programa de Pós Graduação em Medicina, Rua Vergueiro 235/249, São Paulo, CEP 01504-001 Brazil; 2 Heart Institute (InCor), Hospital das Clínicas da Faculdade de Medicina da Universidade de São Paulo, São Paulo, Brazil

**Keywords:** Metabolic syndrome, Clustering, Pulse wave velocity, Arterial stiffness

## Abstract

**Background:**

Metabolic syndrome (MetS) is associated with structural and functional vascular abnormalities, which may lead to increased arterial stiffness, more frequent cardiovascular events and higher mortality. However, the role played by clustering of risk factors and the combining pattern of MetS risk factors and their association with the arterial stiffness have yet to be fully understood. Age, hypertension and diabetes mellitus seem to be strongly associated with increased pulse wave velocity (PWV). This study aimed at determining the clustering and combining pattern of MetS risk factors and their association with the arterial stiffness in non-diabetic and non-hypertensive patients.

**Methods:**

Recently diagnosed and untreated patients with MetS (n = 64, 49 ± 8 year, 32 ± 4 kg/m^2^) were selected, according to ATP III criteria and compared to a control group (Control, n = 17, 49 ± 6 year, 27 ± 2 kg/m^2^). Arterial stiffness was evaluated by PWV in the carotid-femoral segment. Patients were categorized and analyzed according MetS risk factors clustering (3, 4 and 5 factors) and its combinations.

**Results:**

Patients with MetS had increased PWV when compared to Control (7.8 ± 1.1 vs. 7.0 ± 0.5 m/s, p < 0.001). In multivariate analysis, the variables that remained as predictors of PWV were age (β = 0.450, p < 0.001), systolic blood pressure (β = 0.211, p = 0.023) and triglycerides (β = 0.212, p = 0.037). The increased number of risk factors reflected in a progressive increase in PWV. When adjusted to systolic blood pressure, PWV was greater in the group with 5 risk factors when compared to the group with 3 risk factors and Control (8.5 ± 0.4 vs. 7.5 ± 0.2, p = 0.011 and 7.2 ± 0.3 m/s, p = 0.012). Similarly, the 4 risk factors group had higher PWV than the Control (7.9 ± 0.2 vs. 7.2 ± 0.3, p = 0.047).

**Conclusions:**

The number of risk factors seems to increase arterial stiffness. Notably, besides age and increased systolic blood pressure, alterations in the triglycerides worsened the stiffness of large vessels, emphasizing the importance in addressing this risk factor in MetS patients.

## Background

Metabolic syndrome (MetS) is characterized by a clustering of abnormalities such as central obesity, insulin resistance, dyslipidemia and arterial hypertension [1, 2]. Since it has been strongly associated with increased risk of cardiovascular disease, type 2 diabetes, and increased mortality, MetS is now recognized as a major public health issue [3–5].

Although each risk factor alone carries its own impact on health, risk factors are often found clustered in individuals [6, 7]. MetS can be defined by the presence of three to five metabolic components, allowing various possibilities of combinations that share some basic pathophysiological patterns [8]. Studies have shown that both, the number and the interaction of different combinations of risk factors, may cause variations in cardiovascular mortality risk [8, 9].

Each risk factor acts independently through different mechanisms, causing negative alterations in the structure and function of the vascular system [10, 11]. Hypertension involves hemodynamic and overload mechanisms that reflect changes in the extracellular matrix, affecting the structural remodeling of the vessels [12]. It also determines a significant increase in arterial resistance by the accumulation of collagen, induced both by sodium and by alterations in the renin angiotensin-aldosterone system [12].

Hyperinsulinemia and chronic hyperglycemia may increase the activity of the sympathetic nervous system, the vascular inflammation, and the activity of renin–angiotensin–aldosterone system in vascular tissue, causes the development of wall hypertrophy and fibrosis [13]. In addition, inadequate advanced glycation end-products in arterial wall may cause significant changes in elastin with the accumulation of collagen, thus reducing the distensibility of the vessel receptor [13].

It has been reported that, in the general population, MetS accelerates arterial aging. Individuals with MetS have twofold risk of presenting stiff arteries when compared to individuals without MetS, even after controlling for age, sex and occurrence of diabetes mellitus [14]. Arterial stiffness of central arterial segments is normally identified before clinical manifestations of cardiovascular disease [15].

Arterial stiffness can be measured by pulse wave velocity (PWV), which is a simple, sensitive and non-invasive method. PWV is used for diagnosis and prognosis in patients at risk for atherosclerotic cardiovascular disease, and is thus considered an independent predictor of cardiovascular mortality [16–19].

It is well-established that age and hypertension are the main causes of arterial stiffness [20]. Additionally, Hwang et al. [21] have found that, regardless of age and blood pressure (BP), subjects with diabetes mellitus had higher PWV, whereas subjects with prediabetes had similar PWV when compared to subjects with normal fasting blood glucose. Moreover, we have recently observed that small changes in fasting glycaemia in MetS patients free of diabetes and hypertension are associated with vascular impairment [22].

However, few studies have addressed the complexity of clustering and patterns of combination of MetS risk factors in vascular dysfunction [23, 24]. It is not known in non-diabetic and non-hypertensive patients, but with 3, 4 or 5 MetS components, the role of each MetS risk factor as well as the role of clustering and the combining pattern components in arterial stiffness [24, 25].

Further studies are needed to elucidate the mechanisms by which MetS increases cardiovascular risk. A fuller understanding of such mechanisms would benefit clinical practice and allow targeted interventions in preventive and therapeutic strategies in the early stage of cardiovascular disease [26, 27].

Our hypothesis was that in early stages of MetS, patients already present arterial stiffness. And, considering the cluster of MetS risk factors, we aimed to determine which of these factors are better predictors of this alteration.

Then, the objective of this study was to analyze the clustering and combining pattern of MetS risk factors, as well as the associations of each component of MetS and arterial stiffness.

## Methods

### Study population

This is a cross-sectional study conducted at the Heart Institute (InCor), at the University of São Paulo Medical School, São Paulo State, Brazil. The study was approved by the Scientific Commission of the Heart Institute (InCor), and by the Ethics Research Commission of the Hospital das Clínicas de São Paulo. Each patient signed a written consent form.

After being diagnosed with MetS, outpatients from the Cardiovascular Rehabilitation and Exercise Physiology Ambulatory of the Heart Institute (InCor), University of Sao Paulo Medical School, were invited to participate in the study.

Individuals were diagnosed with MetS according to the operational criteria of the “National Cholesterol Education Program, Adult Treatment Panel III (ATP III)” [28] with at least 3 of the 5 MetS risk factors present: (1) waist circumference (WC) ≥ 102 cm for men and ≥ 88 cm for women, (2) triglycerides ≥ 150 mg/dL, (3) high-density lipoprotein cholesterol (HDL-c) < 40 mg/dL for men and < 50 mg/dL for women, (4) systolic blood pressure (SBP) ≥ 130 mmHg or diastolic blood pressure (DBP) ≥ 85 mmHg, and (5) blood glucose ≥ 100 mg/dL. The patients were eligible for enrollment if they met the following inclusion criteria: sedentary with no regular physical activity (> 3 days/week, > 30 min/day) for the previous 6 months, aged between 30 and 60 years, non-smokers, without a history of excessive alcohol consumption, with no evidence of pulmonary or cardiovascular disease, and not taking any medications. Patients with hypertension and/or diabetes were excluded. The control group (Control) consisted of participants without any risk factor or with only one risk factor, following the same criteria used for the MetS group.

### Study design

The MetS risk factors were categorized in the clustering of 3, 4 or 5 risk factors. In addition, the combining pattern of risk factors were analyzed. The groups were compared to Control with similar demographic characteristics.

### MetS risk factors measurements

Waist circumference was measured at the end of normal expiration to the nearest 0.1 cm, measuring from the narrowest point between the lower borders of the rib cage and the iliac crest. In both, three consecutive measurements were undertaken by the same evaluator, and the average value of the measurements was recorded. Also, body mass index (BMI) was calculated through the anthropometric measurement of weight and height [29].

The BP was taken with the subject sitting with legs uncrossed and feet flat on the floor, using a sphygmomanometer (cuff according to the arm size) and a stethoscope on the on the left arm placed at heart level [30].

Laboratory tests were performed in the mornings after a 12 h fasting. Serum levels of total cholesterol, triglycerides and HDL-c were obtained by enzymatic colorimetric method, while glucose was measured by the automated enzymatic method—Roche (oxidase standard glucose).

### Arterial stiffness assessment

Arterial stiffness was assessed by PWV in carotid-femoral segment using a noninvasive automatic device, Complior (Colson, Garges les Gonesses, France), which allows an online pulse wave recording and the automatic calculation of PWV.

Measurements were obtained by positioning two pressure-sensitive transducers (Fukuda, Tokyo, Japan) on to the skin prominent parts of the carotid and femoral arteries to calculate time delay between the two transducers. The distance traveled by the pulse wave was measured over the body surface as the distance between the two recording sites (D), while pulse transit time (t) was measured between the feet of the pressure waveforms recorded at these different points (foot-to-foot method) and was automatically calculated by the device [31]. Measurements were repeated over 10 different cardiac cycles, and the mean was used for the final analysis. PWV was automatically calculated as PWV = D/t, and the values were then standardized to the ‘real’ carotid-femoral distance by multiplying by 0.8, following international recommendations [32].

Measurements were performed in a quiet room with stable room temperature and the patient in a supine position. During PWV measurements, a continuous noninvasive BP recording was performed using the Portapres device (TNO Biomedical Instrumentation, Amsterdam, The Netherlands). The mean of three stable measurements was used for final analysis.

### Statistical analysis

Sample size was determined by OPEN EPI [33] with PWV considered as the main outcome variable. A 80% power, with a 2-tailed type I error of 0.05, was necessary to detect the differences in arterial stiffness between groups (MetS vs. C). For this case were required 19 participants for group.

The normality of data was determined using the Kolmogorov–Smirnov test. The descriptive measures were obtained by absolute and relative frequency distribution and included both central trend calculations (mean) and dispersion (standard deviation). For inferential analysis, the Chi square test was used to analyze the sex distribution in each studied groups. The groups comparison analyzes for independent samples and Levine test were performed for variable studied as follows: Student’s t test for the physical, metabolic data between Control vs. MetS group; one-way analysis of variance (ANOVA) followed by Scheffé post hoc for the physical, metabolic data between Control and clustering of risk factors groups; and analysis of covariance (ANCOVA) adjusting the SBP variable for the PWV between Control and clustering of risk factors groups. Pearson correlation (r) was also performed between PWV and age, each MetS risk factors, and for variables with p < 0.05 a multivariate linear regression (stepwise regression—forward selection) was performed to determine the predictive variables of PWV.

Statistical analysis was performed using the Statistical Package for the Social Sciences for Windows (SPSS, version 20.0), and the level of significance adopted for all statistical analysis was p < 0.05.

## Results

Sixty-four patients (n = 35, 55% women) were diagnosed with MetS in the current study. They were allocated into three groups according to the clustering of MetS: (1) 5 risk factors (n = 8); (2) 4 risk factors (n = 27); and (3) 3 risk factors (n = 29). In addition, these 3 groups were compared to control subjects without MetS (Control, n = 17).

Variables were normally distributed, and descriptive measurements are presented as mean ± standard deviation. Baseline measurements are presented in Table 1. There were no differences in sex distribution and in age among studied groups. Regardless of the number of risk factors, MetS groups had higher BMI, WC, triglycerides, SBP and DBP and lower HDL-c than the Control. Blood glucose was higher only in the 5 and 4 risk factors groups when compared to Control (Table 1). Triglycerides were higher only in the 5 risk factors group when compared with the 3 risk factors group (Table 1). It should be noted that WC was the most prevalent risk factor and HDL-c was the second risk factor with high prevalence in all groups.

**Table 1:** Characteristics of groups in clustering of risk factors for MetS

Characteristics	Total MetS (n = 64)	5 factors (n = 8)	4 factors (n = 27)	3 factors (n = 29)	Control (n = 17)
Female sex (n/%)	35/55	03/38	15/56	17/59	11/65
Age (years)	49 ± 8	54 ± 8	49 ± 8	47 ± 7	49 ± 6
BMI (kg/m^2^)	32 ± 4*	32 ± 2*	32 ± 5*	32 ± 3*	27 ± 2
MetS risk factors and prevalence					
WC (cm)	106 ± 9* 94%	109 ± 6* 100%	107 ± 11* 96%	104 ± 7* 90%	93 ± 7 47%
TG (mg/dL)	178 ± 78* 59%	250 ± 85*^†^ 100%	183 ± 68* 67%	154 ± 75* 41%	92 ± 59 6%
HDL-c (mg/dL)	41 ± 10* 80%	35 ± 3* 100%	39 ± 7* 93%	45 ± 12* 62%	59 ± 16 18%
BG (mg/dL)	103 ± 10* 59%	107 ± 4* 100%	105 ± 11* 74%	100 ± 12 35%	92 ± 7 18%
SBP (mmHg)	129 ± 13* 58%	136 ± 15* 88%	128 ± 14* 52%	128 ± 13* 55%	111 ± 7 0%
DBP (mmHg)	84 ± 11* 55%	93 ± 9* 88%	81 ± 11* 44%	84 ± 11* 55%	71 ± 9 6%

Mean value ± standard deviation

*MetS* metabolic syndrome, *BMI* body mass index, *WC* waist circumference, *TG* triglycerides, *HDL-c* high-density lipoprotein cholesterol, *BG* blood glucose, *SBP* systolic blood pressure, *DBP* diastolic blood pressure

* p < 0.05 vs. control

^†^ p < 0.05 vs. 3 risk factors

Descriptive measures of vascular function by PWV in the combinations of risk factors in MetS patients are shown in Table 2. Regardless of the number of risk factors 3, 4 (plus HDL-c) or 5, the most prevalent combination of risk factors in all groups was increased blood glucose, BP and WC.

**Table 2:** Pulse wave velocity in risk factors combination of the MetS

Risk factors combination	MetS (n = 64)			
	n	%	PWV (m/s)	SD
5				
BG + TG + BP + WC + HDL-c	8	12.5	8.7	1.5
4				
BG + BP + WC + HDL-c	9	14.1	7.5	0.9
BG + TG + WC + HDL-c	8	12.5	8.6	1.5
TG +BP + WC + HDL-c	7	10.9	7.8	1.4
BG + TG + BP + WC	2	3.1	8.1	1.4
BG + TG + BP + HDL-c	1	1.6	8.6	–
3				
BG + BP + WC	8	12.5	7.6	1.0
BP + WC + HDL-c	7	10.9	7.3	0.4
TG + WC + HDL-c	6	9.4	7.7	1.1
TG + BP + WC	3	4.7	7.3	0.3
TG + BP + HDL-c	3	4.7	7.1	0.9
BG + WC + HDL-c	2	3.1	7.7	1.1
BG + TG + BP	–	–	–	–
BG + TG + WC	–	–	–	–
BG + TG + HDL-c	–	–	–	–
BG + BP + HDL-c	–	–	–	–

Mean value and standard deviation (SD)

*MetS* metabolic syndrome, *PWV* pulse wave velocity, *BG* blood glucose, *TG* triglycerides, *BP* blood pressure, *WC* waist circumference, *HDL-c* high-density lipoprotein cholesterol

Univariate linear regression analysis showed a positive association between PWV in carotid-femoral segment and age, triglycerides, SBP and blood glucose, while presenting a negative association with HDL-c (Table 3). No association was found between PWV and either WC or DBP. For multivariate linear regression, the variables with p < 0.05 were included. The variables which remained as predictors of PWV in carotid-femoral segment were age, SBP and triglycerides (Table 3).

**Table 3:** Univariate and multivariate linear regression between PWV (dependent variable) and age and MetS risk factors

	Univariate linear regression		Multivariate linear regression		
	r/r^2^	p	β	(95% CI)	p
Dependent variable: PWV					
Age (years)	0.486/0.236	< 0.001	0.450	(0.038 to 0.091)	< 0.001
WC (cm)	0.215/0.046	0.055	–	–	–
TG (mg/dL)	0.374/0.140	< 0.001	0.212	(0.000 to 0.005)	0.037
HDL-c (mg/dL)	− 0.269/0.072	0.015	− 0.187	(− 0.031 to 0.001)	0.066
BG (mg/dL)	0.225/0.050	0.044	0.034	(− 0.015 to 0.022)	0.714
SBP (mmHg)	0.336/0.113	0.002	0.211	(0.002 to 0.030)	0.023
DBP (mmHg)	0.157/0.025	0.161	–	–	–

*MetS* metabolic syndrome, *WC* waist circumference, *TG* triglycerides, *HDL-c* high-density lipoprotein cholesterol, *BG* blood glucose, *SBP* systolic blood pressure, *DBP* diastolic blood pressure, *CI* confidence interval

**Fig. 1:**
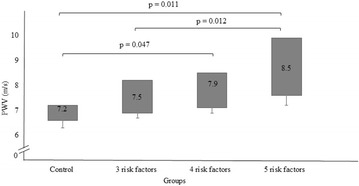
Pulse wave velocity (PWV) of clustering MetS groups. Values inside the blocks are mean values (m/s)

The increase in the number of risk factors caused a progressive impairment in PWV (Fig. 1). When adjusted to SBP measured during the evaluation, PWV was greater in the 5 risk factors group, compared to the 3 risk factors group and Control (8.5 ± 0.4 vs. 7.5 ± 0.2 and 7.2 ± 0.3 m/s, respectively, p < 0.05). Similarly, the 4 risk factors group had higher PWV than the Control (7.9 ± 0.2 vs. 7.2 ± 0.3, p = 0.047).

## Discussion

The main finding in this study lies in the severity of the arterial stiffness in middle-aged individuals, who are in the transition from increasing body weight to established diseases such as hypertension and type 2 diabetes. This transition usually begins with some minor changes, but once combined with a set of risk factors, MetS sets in. These individuals consider themselves “healthy”; however, they frequently present visceral obesity associated with alterations such as a small increase in BP, fasting glucose and/or dyslipidemia. Our results corroborate previous studies, which found that age and increased SBP are important predictors of arterial stiffness [20]. Interestingly, in the present study we found that triglycerides were also predictors of vascular dysfunction, as measured by PWV in MetS patients.

Several previous studies [24, 25] with large population samples have addressed MetS components and arterial stiffness. However, they included patients with cardiovascular disease and/or clinical diabetes. Participants were found to have MetS if they met ATP III, International Diabetes Federation or American Heart Association criteria; these studies defined MetS as the presence of three or more of the five components, and did not distinguished changed levels of glucose and BP and established diabetes and/or hypertension [24, 25]. In addition, they included participants undergoing drug treatments (e.g., anti-hypertensive, hypoglycemic or lipid-lowering drugs), and this might have affected their findings. The difference in our work lies in the sample selection of participants, since we excluded patients with established hypertension and type 2 diabetes, as well as those undergoing drug treatment.

Regardless of the presence of any conditions, the association between aging and the vascular physiological deterioration of the large arteries has been found in several studies [34, 35]. Moreover, linear increase in arterial stiffness has been demonstrated with age [36]. In a study carried out by Tolezani et al. [37] PWV values were increased by 1.3 times from the third to the sixth decade of life, thus lending support to age as a predictor variable, as demonstrated in the present study.

Additionally, most studies have shown a strong association between BP levels and the pathophysiology of arterial stiffness [38, 39]. An European multicenter survey with 11.092 patients has found that patients with BP < 130/85 mmHg without additional cardiovascular risk factors (dyslipidemia and/or smoking) had lower PWV values and showed a lower increase in PWV with age, when compared to other patients with BP ≥ 130/85 mmHg [40]. Also, the survey have indicated that individuals with normal BP values (120–129 and/or 80–84 mmHg) had already higher PWV than subjects with optimal BP (< 120/80 mmHg) [40].

Lipid profile was related to functional and structural parameters of the large arteries, and triglycerides were found to be determinants of vascular parameters for PWV, as well as the distensibility of the carotid artery [37].

Kanga et al. [41] have also shown that aging, lower HDL-c and high triglycerides are also significantly associated with increased PWV in the four groups: control, hypertensive group, MetS without the hypertension risk factor, and MetS group with the presence of hypertension. In their study, SBP was not associated with PWV in any of the MetS groups (normotensive and hypertensive), thus emphasizing the role of the other components of MetS in the pathogenesis of arterial stiffness.

The relationship between lipid profile and arterial stiffness has not yet been well established [24]. However, the researchers indicate that these factors involve concomitant mechanisms such as the development of atherosclerotic plaques, oxidative stress, local and systemic inflammation, endothelial dysfunction and low bioavailability of nitric oxide [13, 37].

The determination of pathophysiological mechanisms underlying the association between PWV and Mets risk factors are beyond the scope of the present study. We might speculate that the fact that the MetS patients had near normal levels of HDL-c and fasting glucose could account for the lack of association between these specific risk factors.

For example, insulin, in physiological concentrations, has been known to act as a vasodilator and as such stimulates the endothelial production of nitric oxide and decreases peripheral resistance [42, 43]. This might account for the lack association between PWV and the fasting glucose alterations observed in the present study. However, in the chronic phase, hyperinsulinemia may increase the activity of the sympathetic nervous system, activate the renin–angiotensin–aldosterone system, and increase systemic and vascular inflammation. In addition, the accumulation of collagen due to inadequate enzymatic glycation in the arterial wall may lead to loss of elasticity, and in turn increasing in arterial stiffness [41, 44]. This may explain the association between PWV and fasting glycaemia in some previous studies [45, 46].

Further investigations addressing the risk threshold between triglycerides and arterial stiffness are needed [24], in order to determine whether the values accepted as normal (< 150 mg/dL), as proposed by the guideline ATP III [28] should be maintained. For example, in 2003 the American Diabetes Association [47] proposed to reduce the cutoff point for the diagnosis of altered fasting glycemia from 110 to 100 mg/dL and the International Diabetes Federation [48] defined new cutoff points for the WC for each ethnic group. The new cutoff points were then incorporated into the definition of MetS, thus allowing earlier interventions in the treatment of these risk factors.

The pathophysiology that determines the increased arterial stiffness in subjects with MetS is yet to be fully understood, and since MetS involves concurrent risk factors, the abnormalities may have a synergistic effect on the damage of the arterial stiffness [41]. For instance, specific clusters of MetS components rather than simply the MetS have been associated with accelerated arterial aging and with cardiovascular events [49, 50].

Vyssoulis et al. [42] have shown that the higher the clustering of MetS risk factors, the greater the impairment in BP level, glycemia, triglycerides and HDL-c. Furthermore, Grundy et al. [51] the magnitude of the increased risk may vary depending on the risk factor present. The main variations in mortality may be masked when MetS is referred to without distinction of their components and their combinations [8].

In the present study, we observed a gradual increase in PWV of the carotid-femoral segment depending on the number of MetS risk factors. Another interesting finding was that the most prevalent MetS risk factor was the increase WC for all groups and that the most prevalent combining pattern in patients with 3, 4 (plus HDL-c) or 5 MetS risk factors was increased blood glucose, BP and WC.

The use of convenience sample is a limitation of our study. We should also point other limitations in the present study. First, our results corroborate previous studies regarding the association between both age and increased SBP with arterial stiffness [20]. However, besides age and SBP, triglycerides were also found to be predictors of PWV in carotid-femoral segment in MetS patients after the multivariate analysis. Secondly, we had a small sample size for identification of the combining patterns of the MetS risk factors. It should be stressed that our selected sample consisted of patients recently diagnosed with MetS, not yet undergoing any drug treatment, non-hypertensive and non-diabetic. Further investigations with larger samples are needed to adequately address these issues.

So, our findings suggest that individuals without cardiovascular disease even if in the early stages of metabolic alterations show increased PWV when compared the control groups. The risk is potentiated with an increase in the number of risk factors for MetS. Besides age and increased SBP, the presence of triglycerides in the clustering, which may lead to a greater impairment in the stiffness of the large vessels, thus demonstrating the importance of identifying individuals at subclinical vascular damage.

## Conclusions

The severity of arterial stiffness was found to depend on the number of risk factors. Alterations in SBP and triglycerides in the clustering may lead to a greater impairment in the stiffness of the large vessels, emphasizing the relevance of early treatment of these risk factors for MetS. This may contribute to inform effective therapies in the treatment of MetS, minimizing the damage to vascular function, and consequently cardiovascular risk.

## Abbreviations

MetS: metabolic syndrome
ATP III: National Cholesterol Education Program—Adult Treatment Panel III
InCor: Heart Institute
SPSS: Statistic Package for the Science Social
ANOVA: analysis of variance
ANCOVA: analysis of covariance
PWV: pulse wave velocity
BMI: body mass index
WC: waist circumference
TG: triglycerides
HDL-c: high-density lipoprotein cholesterol
SBP: systolic blood pressure
DBP: diastolic blood pressure
BP: blood pressure

## References

[1] Parapid B, Ostojic MC, Lalic NM, Micic D, Damjanovic S, Bubanja D (2014). Risk factors clustering within the metabolic syndrome: a pattern or by chance?. Hellenic J Cardiol..

[2] Sungwacha JN, Tyler J, Longo-Mbenza B, On’Kin JBKL, Gombet T, Erasmus RT (2013). Assessing clustering of metabolic syndrome components available at primary care for Bantu Africans using factor analysis in the general population. BMC Res Notes..

[3] Kaur J (2014). A comprehensive review on metabolic syndrome. Cardiol Res Pract..

[4] Mottillo S, Filion KB, Genest J, Joseph L, Pilote L, Poirier P (2010). The metabolic syndrome and cardiovascular risk a systematic review and meta-analysis. J Am Coll Cardiol.

[5] Gami AS, Witt BJ, Howard DE, Erwin PJ, Gami LA, Somers VK (2007). Metabolic syndrome and risk of incident cardiovascular events and death: a systematic review and meta-analysis of longitudinal studies. J Am Coll Cardiol.

[6] Costa FF, Montenegro VB, Lopes TJA, Costa EC (2011). Combinação de fatores de risco relacionados à síndrome metabólica em militares da marinha do Brasil. Arq Bras Cardiol.

[7] Aizawa Y, Watanabe H, Ramadan MM, Usuda Y, Watanabe T, Sasaki S (2007). Clustering trend of components of metabolic syndrome. Int J Cardiol.

[8] Kuk JL, Ardern CI (2010). Age and sex differences in the clustering of metabolic syndrome factors: association with mortality risk. Diabetes Care.

[9] Guize L, Thomas F, Pannier B, Bean K, Jego B, Benetos A (2007). All-cause mortality associated with specific combinations of the metabolic syndrome according to recent definitions. Diabetes Care.

[10] Shimabukuro M, Higa N, Masuzaki H, Sata M, Ueda S (2016). Impact of individual metabolic risk components or its clustering on endothelial and smooth muscle cell function in men. Cardiovasc Diabetol..

[11] Weng C, Yuan H, Tang X, Huang Z, Yang K, Chen W (2012). Age- and gender dependent association between components of metabolic syndrome and subclinical arterial stiffness in a Chinese population. Int J Med Sci..

[12] Pizzi O, Brandão AA, Magalhães MEC, Pozzan R, Brandão AP (2006). Velocidade de onda de pulso—o método e suas implicações prognósticas na hipertensão arterial. Rev Bras Hipertens..

[13] Zieman SJ, Melenovsky V, Kass DA (2005). Mechanisms, pathophysiology, and therapy of arterial stiffness. Arterioscler Thromb Vasc Biol.

[14] Scuteri A, Najjar SS, Orru M, Usala G, Piras MG, Ferrucci L (2010). The central arterial burden of the metabolic syndrome is similar in men and women: the SardiNIA Study. Eur Heart J.

[15] Wang A, Su Z, Liu X, Yang Y, Chen S, Wang S (2016). Brachial-ankle pulse wave velocity and metabolic syndrome in general population: the APAC study. BMC Cardiovasc Disord.

[16] Mitchell GF, Hwang SJ, Vasan RS, Larson MG, Pencina MJ, Hamburg NM (2010). Arterial stiffness and cardiovascular events: the Framingham Heart Study. Circulation.

[17] Lee DH, Youn HJ, Chung WB, Choi YS, Lee JM, Park CS (2017). Effects of metabolic syndrome on aortic pulse wave velocity. Clin Hypertens.

[18] Shokawa T, Imazu M, Yamamoto H, Toyofuku M, Tasaki N, Okimoto T (2005). Pulse wave velocity predicts cardiovascular mortality: findings from the Hawaii-Los Angeles-Hiroshima study. Circ J.

[19] Inoue N, Maeda R, Kawakami H, Shokawa T, Yamamoto H, Ito C (2009). Aortic pulse wave velocity predicts cardiovascular mortality in middle-aged and elderly Japanese men. Circ J.

[20] Laurent S, Boutouyrie P, Asmar R, Gautier I, Laloux B, Guize L (2001). Aortic stiffness is an independent predictor of all-cause and cardiovascular mortality in hypertensive patients. Hypertension.

[21] Hwang HS, Ko KP, Kim MG, Kim S, Moon J, Chung WJ, Shin MS, Han SH (2016). The role of abnormal metabolic conditions on arterial stiffness in healthy subjects with no drug treatment. Clin Hypertens..

[22] Rodrigues S, Cepeda FX, Toschi-Dias E, Dutra-Marques Akothirene CB, Carvalho Jefferson C, Costa-Hong Valéria (2017). The role of increased glucose on neurovascular dysfunction in patients with the metabolic syndrome. J Clin Hypertens.

[23] Yoon JH, Park JK, Oh SS, Lee KH, Kim SK, Kim JK (2011). The clustering patterns of metabolic risk factors and its association with sub-clinical atherosclerosis in Korean population. Ann Hum Biol.

[24] Gomez-Sanchez L, Garcia-Ortiz L, Patino-Alonso MC, Recio-Rodriguez JI, Fernando R, Marti R (2016). Association of metabolic syndrome and its components with arterial stiffness in Caucasian subjects of the MARK study: a cross-sectional trial. Cardiovasc Diabetol..

[25] van Herpt TTW, Dehghan A, van Hoek M, Ikram MA, Hofman A, Sijbrands EJG (2016). The clinical value of metabolic syndrome and risks of cardiometabolic events and mortality in the elderly: the Rotterdam study. Cardiovasc Diabetol..

[26] Pimenta AM, Felisbino-Mendes MS, Velasquez-Melendez G (2013). Clustering and combining pattern of metabolic syndrome components in a rural Brazilian adult population. Sao Paulo Med J.

[27] Aekplakorn W, Kessomboon P, Sangthong R, Chariyalertsak S, Putwatana P, Inthawong R (2011). Urban and rural variation in clustering of metabolic syndrome components in the Thai population: results from the fourth National Health Examination Survey 2009. BMC Public Health.

[28] Expert Panel on Detection, Evaluation, and treatment of high blood cholesterol in Adults (2001). Executive summary of the third report of the National Cholesterol Education Program (NCEP) expert panel on detection, evaluation, and treatment of high blood cholesterol in adults (adult treatment panel III). JAMA.

[29] WHO (2008). Waist circumference and waist–hip ratio: report of a WHO expert consultation.

[30] Chobanian AV, Bakris GL, Black HR, Cushman WC, Green LA, Izzo JL (2003). The National High Blood Pressure Education Program Coordinating Committee. Seventh Report of the Joint National Committee on Prevention, Detection, Evaluation, and Treatment of High Blood Pressure. Hypertension.

[31] Rhee MY, Lee HY, Park JB (2008). Measurements of arterial stiffness: methodological aspects. Korean Circ J..

[32] Bortel LMV, Laurent S, Boutouyrie P, Chowienczyk P, Cruickshank JK, Backera T (2012). Expert consensus document on the measurement of aortic stiffness in daily practice using carotid-femoral pulse wave velocity. J Hypertens.

[33] Dean AG, Sullivan KM, Soe MM. OpenEpi: open source epidemiologic statistics for public health; 2013. http://www.openepi.com.

[34] Diaz A, Galli C, Tringler M, Ramirez A, Cabrera Fischer EI (2014). Reference values of pulse wave velocity in healthy people from an urban and rural argentinean population. Int J Hypertens..

[35] Li S, Chen W, Srinivasan SR, Berenson GS (2005). Influence of metabolic syndrome on arterial stiffness and its age-related change in young adults: the Bogalusa Heart Study. Atherosclerosis..

[36] Pereira T, Maldonado J, Polónia J, Silva JA, Morais J, Marques M (2011). Definição de valores de referência da velocidade da onda de pulso arterial numa população portuguesa: uma sub-análise do projecto EDIVA. Rev Port Cardiol.

[37] Tolezani EC, Costa-Hong V, Correia G, Mansur AJ, Drager LF, Bortolotto LA (2014). Determinants of functional and structural properties of large arteries in healthy individuals. Arq Bras Cardiol.

[38] Li CI, Kardia SLR, Liu CS, Lin WY, Lin CH, Lee YD (2011). Metabolic syndrome is associated with change in subclinical arterial stiffness—a community-based Taichung Community Health Study. BMC Public Health..

[39] Dao HH, Essalihi R, Bouvet C, Moreau P (2005). Evolution and modulation of age-related medial elastocalcinosis: Impact on large artery stiffness and isolated systolic hypertension. Cardiovasc Res.

[40] Mattace-Raso FUS, Hofman A, Verwoert GC, Witteman JCM, Wilkinson I, Cockcroft J (2010). Determinants of pulse wave velocity in healthy people and in the presence of cardiovascular risk factors: ‘establishing normal and reference values. Eur Heart J.

[41] Kangas P, Tikkakoski AJ, Tahvanainen AM, Leskinen MH, Viitala JM, Kahonen M (2013). Metabolic syndrome may be associated with increased arterial stiffness even in the absence of hypertension: a study in 84 cases and 82 controls. Metabolism..

[42] Vyssoulis GP, Pietri PG, Karpanou EA, Vlachopoulos CV, Kyvelou SM, Spanos P (2010). Differential impact of metabolic syndrome on arterial stiffness and wave reflections: focus on distinct definitions. Int J Cardiol.

[43] Bahia L, Aguiar LGK, Villela NR, Bottino D, Bouskela E (2006). O endotélio na síndrome matabólica. Arq Bras Endocrinol Metab..

[44] Ribeiro FA, Thoen RH, Kohler I, Danzmann LC, Torres MAR (2012). Síndrome metabólica: complacência arterial e a velocidade de onda de pulso. Revista da AMRIGS..

[45] Bruno RM, Penno G, Daniele G, Pucci L, Lucchesi D, Stea F (2012). Type 2 diabetes mellitus worsens arterial stiffness in hypertensive patients through endothelial dysfunction. Diabetologia.

[46] Urbina EM, Gao Z, Khoury PR, Martin LJ, Dolan LM (2012). Insulin resistance and arterial stiffness in healthy adolescents and young adults. Diabetologia.

[47] American Diabetes Association (2010). Diagnosis and classification of diabetes mellitus. Diabetes Care.

[48] Yoo YS, Oh SW (2014). Optimal waist circumference cutoff values for the diagnosis of abdominal obesity in Korean adults. Endocrinol Metab..

[49] Scuteri A, Cunha PG, Rosei EA, Badariere J, Bekaert S, Cockcroft JR (2014). Arterial stiffness and influences of the metabolic syndrome: a cross-countries study. Atherosclerosis..

[50] Scuteri A, Laurent S, Cucca F, Cockcroft J, Cunha PG, Mañas LR (2015). Metabolic syndrome across Europe: different clusters of risk factors. Eur J Prev Cardiol..

[51] Grundy SM, Cleeman JI, Daniels SR, Donato KA, Eckel RH, Franklin BA (2005). Diagnosis and management of the metabolic syndrome: an American Heart Association/National Heart, Lung, and Blood Institute Scientific Statement. Circulation.

